# Impact on analgesia, diaphragmatic function, and recovery between erector spinae plane block versus superior trunk block in arthroscopic shoulder surgery: a randomized controlled trial

**DOI:** 10.1080/07853890.2026.2672843

**Published:** 2026-05-14

**Authors:** Hsin-Yu Yang, Chung-Hsun Chang, Yueh-Hsun Chuang, Chen-Tse Lee, Chia-Wei Lee, Chih-Fan Chen, Feng-Sheng Lin, Chun-Yu Wu

**Affiliations:** a Department of Anesthesiology, National Taiwan University Hospital, Taipei, Taiwan; b Department of Anesthesiology and Pain Medicine, University of Washington, Seattle, WA, USA; c Department of Orthopedic Surgery, National Taiwan University Hospital, Taipei, Taiwan; d College of Medicine, National Taiwan University, Taipei, Taiwan; e Department of Anesthesiology, National Taiwan University Hospital, Hsinchu Branch, Hsinchu, Taiwan

**Keywords:** Arthroplasty, nerve block, phrenic nerve, quality of recovery

## Abstract

**Background:**

Effective analgesia and preservation of diaphragmatic function are key considerations in analgesia for shoulder surgery. The superior trunk block provides analgesia with reduced phrenic nerve involvement, while the erector spinae plane block offers minimal impact on diaphragm motion. This randomized controlled trial compared the analgesic efficacy, impact on diaphragmatic motion, and postoperative recovery between the two blocks.

**Methods:**

Sixty patients undergoing arthroscopic shoulder surgery were randomized to receive either erector spinae plane block or superior trunk block. Primary outcomes were postoperative VAS and changes in diaphragmatic excursion. Secondary outcomes included Quality of Recovery-15 (QoR-15) scores, morphine-equivalent consumption, and the handgrip strength motor blockade.

**Results:**

The superior trunk block resulted in significantly lower dynamic VAS at 1-h postoperatively (0.1 [0.0, 0.2] vs. 5.7 [4.0, 7.6]; *p* < 0.001) and reduced 24-h morphine consumption (7.8 [2.5, 15.0] mg vs. 12.7 [7.5, 17.3] mg; *p* = 0.038) compared to the erector spinae plane block. However, diaphragmatic excursion was better preserved in the erector spinae plane block group (8.37% ± 20.7% vs. −20.09% ± 22.2%; *p* < 0.001), with a lower incidence of partial hemidiaphragm paresis (3.3% vs. 46.7%; *p* < 0.001). At 24 h postoperatively, QoR-15 scores were higher in the superior trunk block group (*p* = 0.047), and no patient in either group developed handgrip motor blockade.

**Conclusions:**

Superior trunk block offers superior early postoperative analgesia and better overall recovery, while erector spinae plane block minimizes diaphragmatic impairment. However, the erector spinae plane block may represent an option only in carefully selected patients at high respiratory risk, acknowledging its significantly poorer early analgesic profile.

## Introduction

Inadequate pain control not only prolongs hospital stays but also hinders postoperative rehabilitation in patients undergoing shoulder surgery [1]. Regional nerve blocks play a crucial role in multimodal analgesic protocols for pain management following arthroscopic shoulder surgery [2,3]. The interscalene brachial plexus block is the most commonly performed technique [2]; however, diaphragm dysfunction remains a significant concern [3]. The superior trunk block, performed at the convergence of the C5 and C6 roots to form the superior trunk, is located further from the brachial plexus and phrenic nerve, and has been increasingly advocated as a phrenic-sparing alternative [4,5]. Studies suggest that superior trunk block provides analgesic effects comparable to interscalene nerve blocks while reducing the risk of phrenic nerve involvement [5]. However, substantial evidence indicates that abnormal diaphragm motion remains common in patients receiving superior trunk block [5–7].

Recent literature has highlighted the successful use of a high-thoracic erector spinae plane block for upper extremity analgesia, including shoulder surgery [8–10]. Given concerns regarding diaphragm motion, erector spinae plane block has been proposed as a technically simple and potentially safer alternative for shoulder surgery [6]. However, comparative studies between the superior trunk block and erector spinae plane block remain limited, particularly regarding their analgesic efficacy, effects on hemidiaphragm motion, and impact on postoperative quality of recovery. To address these gaps, we conducted this randomized controlled trial to evaluate and compare the analgesic efficacy, hemidiaphragm motion, and quality of recovery outcomes of superior trunk block versus erector spinae plane block in patients undergoing arthroscopic shoulder surgery.

## Materials and methods

### Participant recruitment and group allocation

This study is a prospective, single-center, randomized controlled trial with a two-arm parallel group design. The trial received approval from the Research Ethics Committee of National Taiwan University Hospital (reference and approval number: 202301211RINB), and the study protocol was registered and published on ClinicalTrials.gov (NCT05822414; principal investigator: Chun-Yu Wu; date of registry: April 10th 2023). Patients undergoing elective arthroscopic surgery were screened based on the following exclusion criteria: age below 18 or above 85 years, chronic obstructive pulmonary disease diagnosed based on the lung function test, heart failure with New York Heart Association Functional Classification III or IV, estimated glomerular filtration rate < 60 mL/min/1.73 m^2^, chronic opioid use and coagulopathy. We conducted the study in accordance with the Declaration of Helsinki and in adherence to the applicable CONSORT guidelines.

Following written consent, we utilized a computer-generated block randomization sequence to allocate participants into one of two study arms (1:1 ratio). To ensure trial integrity, both the surgical patients and the research assistants evaluating outcomes remained unaware of the specific block administered.

### Anesthesia and nerve block

Standard monitoring (ECG, SpO_2_, noninvasive blood pressure) was applied to all subjects. We induced general anesthesia using weight-based doses of fentanyl, propofol, and rocuronium, followed by sevoflurane maintenance titrated to a Bispectral Index (BIS) range of 40–60. Lung ventilation was standardized with a 6–8 mL/kg tidal volume and EtCO_2_ maintained at 30–35 mmHg. If the pulse or mean arterial blood pressure increased by 20% from the preoperative baseline value, 25 μg bolus fentanyl were administered intravenously. Arthroscopic shoulder surgery was performed by the same surgical team using the same technique, in the beach chair position [11]. To prevent nausea and vomiting, 8 mg ondansetron and dexamethasone 5 mg was administered intravenously. In addition, 30 mg of intravenous ketorolac was administered at the end of the surgery. After extubation, the patients with sufficient spontaneous respiration were taken to the recovery room.

Both erector spinae plane block and superior trunk block were performed after the induction of general anesthesia and before turning into the beach chair position using a high frequency linear ultrasound probe (6–13 MHz, Sonosite X-PORTE Ultrasound system). The erector spinae plane block was performed at the T2 level. After positioning the patient laterally, a high-frequency linear ultrasound transducer was placed longitudinally, 2–3 cm lateral to the T2 spinous process until the transverse process comes into view, with the erector spinae muscle seen above. Using the “in plane” technique, a 23-gauge block needle was inserted into the skin in the caudo-cranial direction until hitting the transverse process, and 5-mL normal saline was injected to confirm location of the needle and for hydro dissection. Once adequate spread of fluid was observed in the correct fascial plane, 30 mL of 0.25% bupivacaine [9] was administered.

The superior trunk block was performed with the patient in the supine position and head turned to contralateral side. The probe was placed transversely at the level of supraclavicular fossa, and gradually moved cranially with the C5-8 roots of brachial plexus visualized. The superior trunk was visualized distal to the convergence of the C5 and C6 nerve roots but proximal to the take-off of the suprascapular nerve [12]. A 23-gauge block needle was then advanced from the lateral to the medial direction with the “in plane” technique, and 15 ml of 0.25% bupivacaine [4,5] was administered adjacent to the lateral border of the superior trunk.

### Evaluation of diaphragm excursion and postoperative respiratory care

Diaphragmatic excursion was measured to assess the effect of the nerve block on the phrenic nerve using sonographic methods described in previous studies [4,13–15]. A low-frequency convex transducer probe (2–5 MHz, Sonosite X-PORTE Ultrasound system) was placed over the anterior subcostal area between the midclavicular and axillary lines of the participant to visualize the posterior aspect of the diaphragm in B-mode. On the right side, the liver served as the acoustic window, while the spleen was used on the left. Visualization of the left hemidiaphragm is more challenging using the anterior subcostal view because the spleen provides a smaller acoustic window, and air in the stomach can cause artifacts. To overcome this, the ultrasound probe was placed along the posterior-axillary line, approximately at the 6th to 9th intercostal space, with a slight clockwise rotation to position the indicator towards the posterior axilla. This produced an intercostal oblique view, allowing for clear visualization of the left hemidiaphragm as a thick hyperechoic line [14,15]. Once the diaphragm has been consistently visualized as a hyperechoic line, the ultrasound was then switched to M-mode, with the M-mode line aligned as perpendicular as possible to the hyperechoic diaphragm line. Participants were instructed to perform deep breathing, and the amplitude of diaphragmatic excursion was measured by placing callipers at the bottom and top of the diaphragmatic inspiratory slope [4,13].

Baseline diaphragmatic excursion was recorded before patients entered the operating room, while postoperative excursion was measured at 1 h after surgery, once the patient had regained a full modified Aldrete score [16]. Complete hemidiaphragm paresis was defined as a greater than 75% reduction in diaphragmatic movement from baseline, while partial paresis was defined as a 25–75% reduction [4]. During the stay at post-anesthetic care unit (PACU), patient was encouraged to breathe deeply. The oxygen was provided if the patient developed symptomatic dyspnea or the oxygen saturation was below 94%.

### Postoperative pain management and outcome assessment

Postoperative outcomes were assessed by an independent research assistant who does not participate in clinical care and the ultrasound assessment. Post-surgical pain was managed *via* a standardized multimodal regimen consisting of scheduled acetaminophen (2 g/day) and celecoxib (400 mg/day), managed by clinicians blinded to the study groups. Supplemental analgesia, including IV morphine boluses and oral tramadol/acetaminophen, was provided as rescue therapy based on patient demand [17] prescribed by independent medical staff who did not participate in the nerve block. Postoperative pain intensity was assessed by using the 10-cm visual analogue scale (VAS, ranging from 0 to 10) at 1-h and 24-h after surgery.

The primary outcomes of this study were [1]: the assessment of the highest difference in dynamic pain scores (pain during movement) between the two study groups within 24 h after surgery, and [2] the differences in perioperative diaphragm excursion between the groups. Secondary outcomes included patient-reported outcomes assessed using the Quality of Recovery-15 (QoR-15) questionnaire to evaluate postoperative recovery [18,19] measured at two time points: the day before surgery and 24 h after surgery. Additional outcomes included cumulative morphine-equivalent consumption at 24 h post-surgery [20] and motor blockade of handgrip strength. Handgrip strength was assessed by asking the patient to squeeze the assessor’s index finger and graded on a three-point scale: 2 = able to squeeze tightly, 1 = loose squeeze, and 0 = unable to exert any squeeze [5]. Handgrip strength was measured at two time points: before surgery (baseline) and 1 h after surgery and the score below 2 was considered significant motor blockade.

### Statistical analysis

As pain scores were expected to be non-normally distributed, the study aimed to detect the reported minimal clinically significant difference in median VAS scores of 11.0 [21] between these two nerve block techniques. Based on previous literature regarding the use of the erector spinae plane block for arthroscopic shoulder surgery [9], a standard deviation (SD) of 2.0 was assumed for the distribution of pain scores. The sample size was calculated using the method proposed by O’Keeffe et al. [22]. This calculation indicated that a total of 48 patients would be required to achieve a statistical power of 0.8 with a two-sided type I error rate of 0.05. For the investigation of nerve block-induced diaphragm effects, we assumed an incidence of partial hemidiaphragm paresis of 50% following the superior trunk block, based on existing literature [4–6]. To detect a 35% reduction in the incidence of partial hemidiaphragm paresis, a sample size of 30 patients per group was required (*α* = 0.05 and power of 80%). Therefore, we enrolled a total of 60 patients in the present study. To account for the dual primary outcomes and to control for the potential inflation of the family-wise Type I error rate, a Bonferroni-adjusted alpha level of 0.025 was used to define statistical significance for the primary endpoints. For all secondary outcomes, a two-sided p-value of < 0.05 was considered statistically significant. The intention-to-treat analysis was performed in the present study.

Data distribution was evaluated for normality through Shapiro–Wilk testing and histogram inspection. We compared categorical variables using chi-square or Fisher’s exact tests, while continuous outcomes were analyzed with independent t-tests or Mann–Whitney U tests, depending on their distribution profile. Repeated measures analysis of variance was conducted to evaluate perioperative changes in QoR-15 scores. Data were summarized as mean ± SD or median (25th to 75th percentile range), as appropriate. Statistical analyses were performed using MedCalc Statistical Software version 23.02 (MedCalc Software Ltd., Ostend, Belgium).

## Results

### Study design and patient characteristics

**Figure 1.**
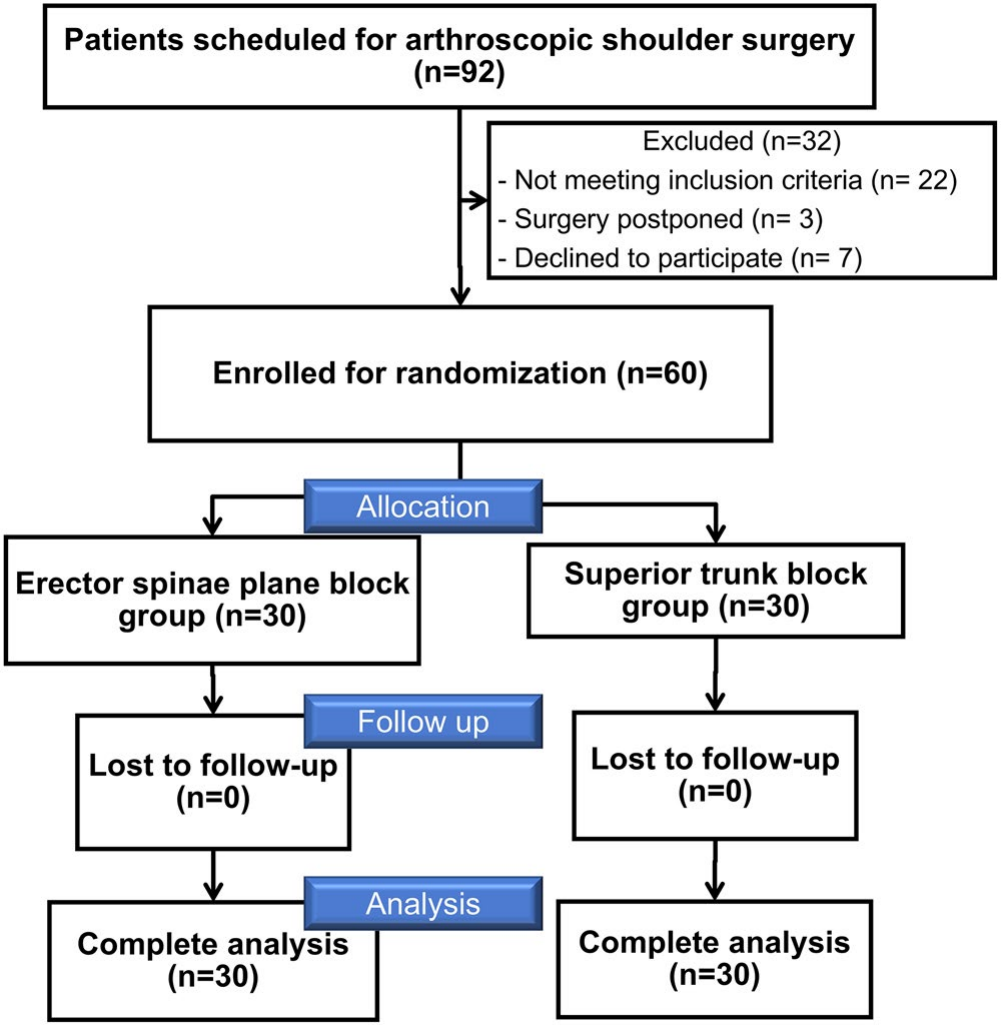
CONSORT diagram. Flowchart illustrating the enrolment, allocation, follow-up, and analysis of participants in the study.

Between 4 May 2023 and 11 December 2024, a total of 60 patients completed the study, with 30 in the erector spinae plane block group and 30 in the superior trunk block group included in the final analysis (Figure 1). The power analysis conducted during the interim analysis yielded power values exceeding 0.9 for the two primary outcomes: the highest VAS differences and the incidence of hemidiaphragm paresis. The two groups were well-balanced in baseline characteristics and surgery types, with all standardized differences within the predefined acceptable range (Table 1).

**Table 1.** Patient characteristics.

	Erector spinae plane block (*n* = 30)	Superior trunk block (*n* = 30)	Standardized difference
*Age (yr)*	66.5 (53.0, 73.0)	64.5 (53.0, 71.0)	0.192
*Gender, male (*n*, %)*	10 (33%)	12 (40.0%)	0.138
*Body mass index (kg/m^2^)*	24.5 ± 3.3	25.1 ± 3.4	0.170
*ASA Classification*			
*Class II (*n*; %)*	22 (73.3%)	22 (73.3%)	0
*Class III (*n*; %)*	8 (26.7%)	8 (26.7%)	0
*Comorbidities (*n*; %)*			
*Hypertension*	11 (36.7%)	10 (33.3%)	0.070
*Arrhythmia*	2 (6.7%)	3 (10.0%)	0.120
*CAD*	2 (6.7%)	0 (0%)	0.378
*Diabetes*	9 (30%)	7 (23.3%)	0.151
*Other*	0	1	0.263
*Right side surgery (*n*; %)*	17 (56.7%)	17 (56.7%)	0
*Type of surgery*			0.350
*Rotator cuff repair*	28 (93.4%)	26 (86.7%)	
*Bankart repair*	1 (3.3%)	3 (10.0%)	
*Capsule reconstruction*	1 (3.3%)	1 (3.3%)	

Values are presented as mean ± *SD* or median (25th percentile, 75th percentile).

### Intraoperative profiles, postoperative analgesic consumption, and pain intensity

Table 2 summarizes intraoperative profiles and postoperative analgesic consumption. There were no significant differences in operation duration or intraoperative hemodynamic parameters, including heart rate and mean arterial pressure, between the two groups. However, patients in the erector spinae plane block group required significantly higher intraoperative fentanyl doses compared to the superior trunk block group [112.5 (100.0, 150.0) µg vs. 100.0 (75.0, 125.0) µg; *p* = 0.028]. Additionally, the erector spinae plane block group had higher postoperative morphine-equivalent consumption at 1 h [4.0 (2.0, 5.0) mg vs. 0.0 (0.0, 0.0) mg; *p* < 0.001] and 24 h [12.7 (7.5, 17.3) mg vs. 7.8 (2.5, 15.0) mg; *p* = 0.038] (Table 2).

**Table 2.** Intraoperative profile and postoperative analgesic consumption.

	Erector spinae plane block (*n* = 30)	Superior trunk block (*n* = 30)	*p* Value
*Operation duration (min)*	99.5 (77.0, 125.0)	116.9 (96.0, 127.0)	0.239
*Intraoperative fentanyl (mcg)*	112.5 (100.0, 150.0)	100.0 (75.0, 125.0)	0.028
*Intraoperative nicardipine (mg)*	0.0 (0.0–1.0)	0.0 (0.0-0.0)	0.161
*Intraoperative hemodynamic*			
*Lowest heart rate, bpm*	60.0 (56.0, 67.0)	62.0 (56.0, 68.0)	0.6045
*Highest heart rate, bpm*	81.7 ± 13.6	85.7 ± 13.1	0.247
*Lowest MAP, mmHg*	64.6 ± 10.4	63.3 ± 9.7	0.6262
*Highest MAP, mmHg*	102.2 ± 10.8	96.5 ± 14.5	0.0917
*Morphine equivalent dose (mg)*			
*1-h after surgery*	4.0 (2.0, 5.0)	0 (0, 0)	<0.001
*24-h after surgery*	12.7 (7.5, 17.3)	7.8 (2.5, 15.0)	0.038
*Number of oral rescue analgesics,* n	1 (0, 4)	1 (0, 4.)	0.920

Values are presented as mean ± *SD* or median (25th percentile, 75th percentile).

**Figure 2.**
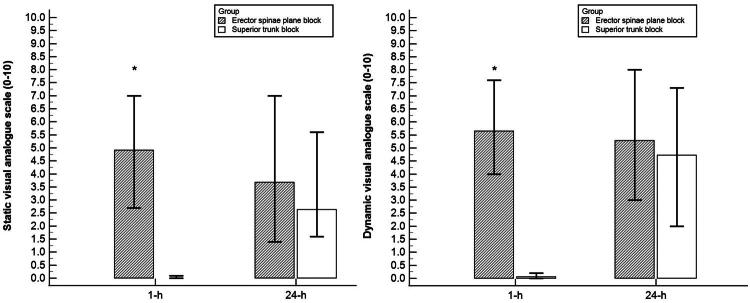
Postoperative pain scores. (A) Postoperative static visual analogue scale (VAS) scores comparing the erector spinae plane block and superior trunk block groups. (B) Postoperative dynamic visual analogue scale (VAS) scores comparing the two groups.

Postoperative pain scores are shown in Figure 2. At 1 h postoperatively, both static and dynamic VAS scores were significantly higher in the erector spinae plane block group than in the superior trunk block group [Static VAS: 4.9 (2.7, 7.0) vs. 0.0 (0.0, 0.1); *p* < 0.001; Dynamic VAS: 5.7 (4.0, 7.6) vs. 0.1 (0.0, 0.2); *p* < 0.001]. At 24 h postoperatively, there were no significant differences in either static or dynamic VAS scores between the two groups.

### Perioperative diaphragm excursion, respiratory profiles, and quality of recovery

**Figure 3.**
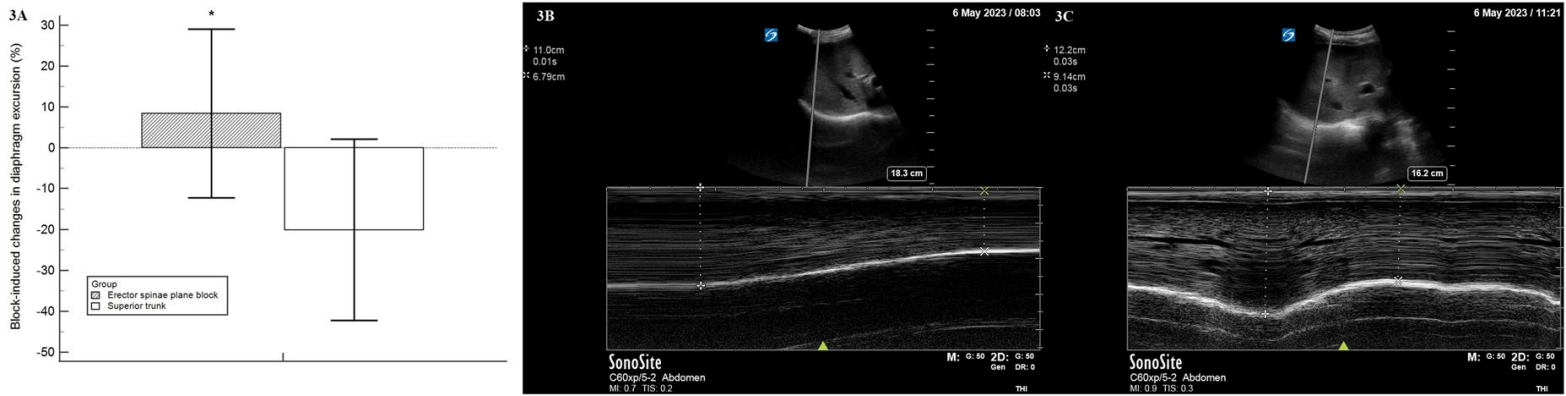
Nerve block-induced diaphragm excursion impacts. (A) Postoperative changes in diaphragm excursion between the erector spinae plane block and superior trunk block groups. (B) Ultrasound illustration of normal diaphragm excursion (4.21 cm) before the superior trunk block. (C) Ultrasound illustration of partial hemidiaphragm paresis, with diaphragm excursion reduced to 2.86 cm, induced by the superior trunk block postoperatively.

Table 3 summarizes nerve block-associated adverse effects, and Figure 3 shows perioperative changes in diaphragm excursion. Baseline diaphragm excursion was similar between groups, but the postoperative reduction in diaphragm excursion was significantly lower in the erector spinae plane block group compared to the superior trunk group [8.37% (20.7%) vs. −20.09% (22.2%); *p* < 0.001; Figure 3A]. Correspondingly, fewer patients in the ESPB group exhibited partial hemidiaphragm paresis than those in the superior trunk block group (3.3% vs. 46.7%; *p* < 0.001; Table 3). No patients in either group exhibited complete hemidiaphragm paresis. Additionally, there were no reports of handgrip strength motor blockade at 1 h postoperatively or requirements for supplemental oxygen during the PACU stay. Oxygen saturation levels, both at the lowest point during PACU stay and at discharge, were comparable between the two groups.

**Table 3.** Perioperative assessments of nerve block associated adverse effects.

	Erector spinae plane block (*n* = 30)	Superior trunk block (*n* = 30)	*p* Value
*Diaphragm excursion, cm*			
*Baseline*	4.1 ± 0.9	4.5 ± 1.0	0.154
*1-h after surgery*	4.4 ± 1.0	3.5 ± 0.9	0.001
*Hemidiaphragm paresis (n; %)*	1 (3.3%)	14 (46.7%)	< 0.001
*Absence/partial/complete paresis, n*	29/1/0	16/14/0	< 0.001
*Handgrip strength blockade, n*			NA
*Baseline*	0	0	
*1-h after surgery*	0	0	
*Additional oxygen support in PACU, n*	0	0	NA
*Oxygen saturation, %*			
*Lowest in PACU*	97 (96, 99)	96 (95, 97)	0.319
*PACU discharge*	98 (97, 100)	98 (96, 99)	0.286
*Duration of PACU stay, min*	65.0 (60.0, 75.0)	60.0 (60.0, 70.0)	0.348

Values are presented as, mean ± SD or median (25th percentile, 75th percentile).

NA: non-applicable; PACU: post-anesthetic care unit.

**Figure 4.**
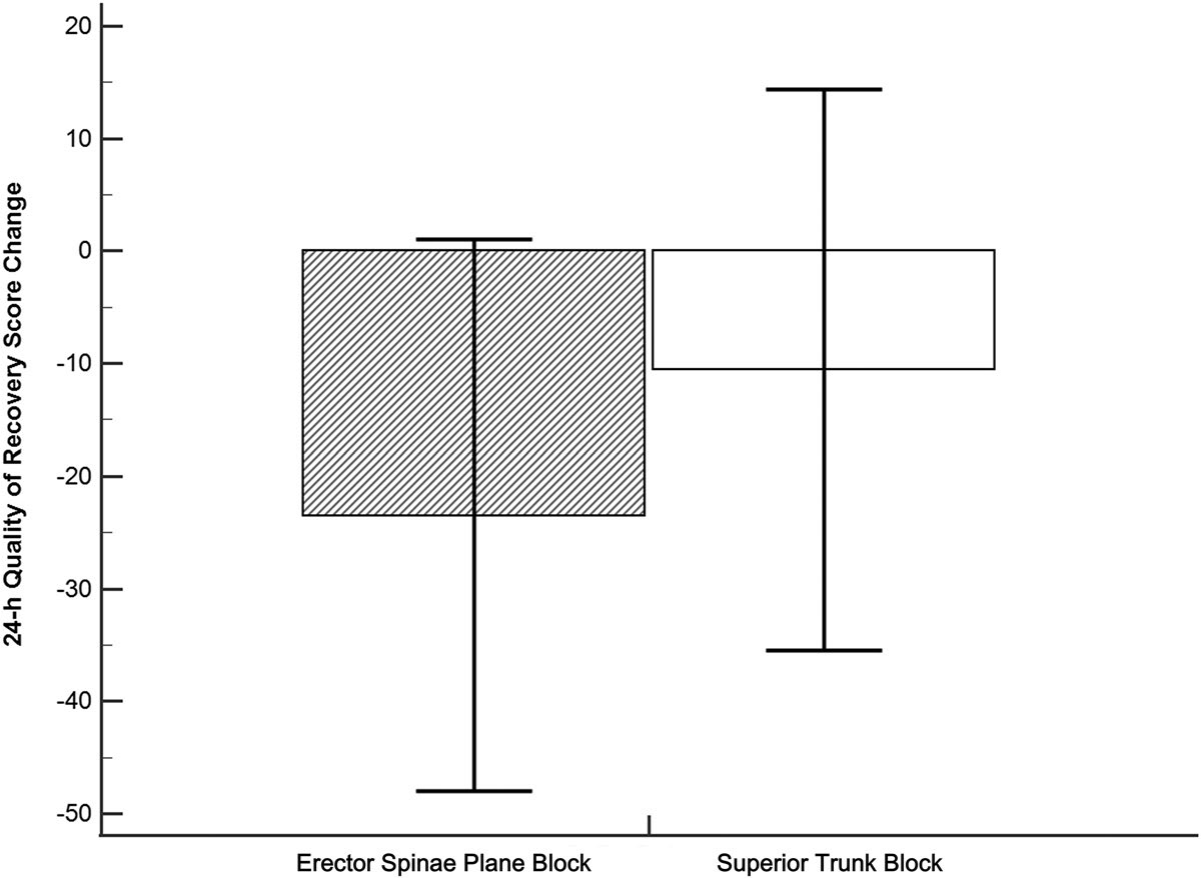
Postoperative reduction in Quality of Recovery-15 (QoR-15) scores. Comparison of reductions in QoR-15 scores between the erector spinae plane block and superior trunk block groups.

Figure 4 shows postoperative changes in QoR-15 scores. Baseline QoR-15 scores were similar between the groups [124.0 ± 20.2 (ESPB) vs. 126.7 ± 15.7 (superior trunk block); *p* = 0.565]. However, the superior trunk block group exhibited significantly smaller reductions in QoR-15 scores at 24 h postoperatively [−23.5 ± 24.5 vs. −10.5 ± 24.9; *p* = 0.047]. Supplementary File 1 provides details of perioperative changes in QoR-15 scores, indicating that differences were primarily due to improved emotional state (question 9: feeling comfortable) and reduced pain severity (question 12: severe pain) in the superior trunk block group.

## Discussion

In the present study, we obtained several notable findings. First, the superior trunk block was associated with more favorable analgesic outcomes, including lower early postoperative pain scores, reduced morphine-equivalent doses, and higher postoperative recovery scores at 24 h. Second, the erector spinae plane block did not affect the range of diaphragm excursion. In contrast, the superior trunk block significantly reduced diaphragm motion, although complete hemidiaphragm paresis was not observed.

In the present study, we observed that the superior trunk block provided adequate analgesic effects, evidenced by a lower intraoperative fentanyl requirement as well as a low median dynamic VAS score below 1 in the PACU, consistent with findings in the literature [4,5]. The interscalene block remains the current standard nerve block technique [2], but the superior trunk block offers several potential advantages. First, the superior trunk is surrounded by a hyperechoic connective tissue sheath, making it easier than the hypoechoic target of interscalene block to identify [5]. Second, the superior trunk block resulted in less reduction in diaphragm movement and higher patient satisfaction in the PACU [4]. However, the effects of the superior trunk block on diaphragm movement have been inconsistently reported in the literature. The reported incidences of partial and complete hemidiaphragm paresis range from 33.3% to 71.1% and 0% to 4.8%, respectively [4–6]. Furthermore, more than one-third of patients with superior trunk block-induced diaphragmatic effects may experience dyspnea despite preserved oxygen saturation [6]. These findings indicate that hemidiaphragm paresis remains a relevant concern with the superior trunk block and may not always be detectable *via* oxygen saturation measurements. In our study, we observed neither objective indicators (e.g. oxygen desaturation or need for supplemental oxygen) nor subjective features (e.g. dyspnea reported in QoR-15 scores) suggestive of superior trunk block-induced hemidiaphragm paresis.

The measurement of quality of recovery after surgery is a key patient-centered outcome. The QoR-15 questionnaire, which includes five domains—physical comfort, emotional state, psychological support, physical independence, and pain—has demonstrated good validity, reliability, and responsiveness across various surgical settings, including orthopedic day-case surgeries [18]. These domains are particularly relevant when comparing nerve block techniques in terms of analgesic efficacy and respiratory impact. In the present study, the superior trunk block group exhibited a significantly smaller reduction in QoR-15 scores at 24 h postoperatively. Notably, the difference in the change of QoR-15 scores between the two groups (13 points) exceeded the established minimal clinically important difference of 6.0–8.0 [23,24], suggesting that the superior trunk block may offer a more favorable early recovery profile compared to the high thoracic erector spinae plane block. However, as the p-value is near the significance threshold, these findings regarding postoperative recovery should be interpreted with caution and warrant further confirmation in larger prospective studies.

The analgesic efficacy of the erector spinae plane block has been repeatedly demonstrated to be superior to sham blocks [10] or no blocks [25] in patients undergoing arthroscopic shoulder surgery. However, the superior analgesic effects of fascial plane blocks may be partially attributed to systemic effects rather than effective blockade of the targeted innervation responsible for postoperative pain [26,27]. Therefore, direct comparisons between erector spinae plane block and brachial plexus blocks are clinically more relevant. In this context, Kapukaya et al. reported that a single-shot erector spinae plane block was associated with worse early postoperative pain scores at 4 h post-surgery compared to the interscalene block, although pain intensity over the longer term was similar between the two techniques [9]. Similarly, Sun et al. found that high thoracic erector spinae plane block was associated with worse pain scores than the interscalene block, but erector spinae plane block resulted in zero incidence of hemidiaphragm paralysis, even with continuous infusion [28]. Our findings align with these observations. Patients in the erector spinae plane block group reported significantly higher pain scores at 1 h post-surgery compared to those in the superior trunk block group. Notably, the median dynamic pain score of approximately 5.7 on a 10-cm VAS in the erector spinae plane block group at 1 h represents a clinically concerning level of pain that may be inadequate for effective early postoperative rehabilitation. For this study, we used a 30 mL injectate for erector spinae plane block, as successful spread into the paravertebral space has been reported to be volume-dependent, with 30 mL being a preferred volume [29,30]. Nonetheless, the injectate spread of the erector spinae plane block is not solely dependent on volume but is influenced by other factors, including the injection site on the transverse process, injection speed, dynamic variations in intra-compartmental pressures, tissue permeability, and potentially other unknown factors [29,31]. This variability in injectate spread may limit the analgesic efficacy of erector spinae plane block in arthroscopic shoulder surgery. Nevertheless, the erector spinae plane block may also benefit patients undergoing prolonged surgery. Prolonged arthroscopic shoulder surgery often involves the use of large volumes of irrigation fluid, which can result in extravasation and edema in the axilla and anterior chest wall. These areas, innervated by the T1 to T2 spinal nerves, are not effectively covered by brachial plexus blocks. Therefore the erector spinae plane block has been reported as a rescue analgesic technique in complex shoulder arthroscopic surgery [32]. However, based on our findings, the erector spinae plane block should not be considered a substitute for brachial plexus block as a primary postoperative analgesic strategy for shoulder surgery.

Although we observed a worse analgesic and recovery profile for the erector spinae plane block compared to the superior trunk block, the risk of diaphragmatic paresis with superior trunk block may be concerning for patients with compromised baseline pulmonary function. This includes individuals with chronic obstructive pulmonary disease, morbid obesity, or obstructive sleep apnea, in whom symptoms might emerge and necessitate treatment [3]. The suprascapular nerve block is another phrenic nerve-sparing alternative to brachial plexus blockade for shoulder surgery. For instance, recent comparisons of the suprascapular nerve block with superior trunk block reported inferior analgesic effects but better preservation of diaphragm motion [33], findings that align with the erector spinae plane block outcomes in our study. Regarding the analgesic efficacy between the suprascapular nerve block and erector spinae plane block, Abdelhaleem et al. observed that erector spinae plane block was associated with lower pain intensity at 2 h post-surgery [34]. Given the current study indicated the inadequate early analgesic effects of erector spinae plane block, both erector spinae plane block and suprascapular nerve block should be considered an option only in carefully selected high respiratory-risk patients.

The present study has several limitations. First, pain score may be optimally assessed at multiple predefined postoperative time points (e.g. 1, 6, 12, and 24 h) for clarification of postoperative pain trajectory between the two blocks. However, we did no have enough research manpower to this sequential analyses in the present study. Second, diaphragm excursion was not evaluated at 24 h post-surgery due to a shortage of research manpower. Consequently, we could not confirm whether the absence of dyspnea symptoms reported in the QoR-15 was due to the diminishing effects of the superior trunk block. Third, phrenic nerve palsy was not assessed using spirometry. However, previous studies have shown that diaphragm ultrasound is more sensitive to unilateral diaphragmatic impairment than spirometry or oxygen saturation, as the latter two methods assess bilateral pulmonary function simultaneously [3]. Fourth, both nerve blocks were performed after the induction of general anesthesia, which may have influenced the primary analgesic outcome measured at 1 h postoperatively. This methodology could have disadvantaged the erector spinae plane block group which required a longer time to establish. However, the duration of surgery in this study population should be sufficient for block onset and would not result in inadequate block duration [29]. Fifth, only the ipsilateral hemidiaphragm was assessed in this study. Since general anesthesia and controlled mechanical ventilation may independently alter diaphragmatic motion, measuring the contralateral hemidiaphragm as an internal control would have strengthened the interpretation of observed changes attributable to the regional technique rather than to residual effects of general anesthesia. Nevertheless, our institutional extubation protocol requires a train-of-four ratio > 0.9, which may partially mitigate the potential confounding effects of residual neuromuscular blockade. Additionally, we did not explore whether a continuous erector spinae plane block could improve analgesic efficacy while maintaining minimal impact on diaphragm excursion.

In conclusion, the present study suggests that the superior trunk block is associated with superior early postoperative analgesia and supports a better early recovery profile compared to the erector spinae plane block in patients undergoing arthroscopic shoulder surgery. However, the erector spinae plane block offers the advantage of preserving diaphragm excursion. The erector spinae plane block may represent an option only in carefully selected patients at high respiratory risk, acknowledging its significantly poorer early analgesic profile. Future studies should further explore the potential applications of erector spinae plane block in this patient population.

## Supplementary Material

Supporting Fiel 1.docx

CONSORT_2025_editable_checklist.docx

## Data Availability

The data that support the findings of this study are available from the corresponding author upon reasonable request. The data are not publicly available because of privacy and ethical restrictions.
