# Supplementary material for: Impact on analgesia, diaphragmatic function, and recovery between erector spinae plane block versus superior trunk block in arthroscopic shoulder surgery: a randomized controlled trial
Source: Ann Med. 2026 May 14;58(1):2672843. doi: 10.1080/07853890.2026.2672843 (PMC13178030; doi:10.1080/07853890.2026.2672843)
Supplement: Supporting Fiel 1.docx [file IANN_A_2672843_SM5746.docx]

Supplementary file 1.

|  | **Erector spinae plane block (n=30)** | **Superior trunk block (n=30)** | **Repeated measured ANOVA group by time interaction P value** |
| --- | --- | --- | --- |
| QoR1: Able to breathe easily | | | |
| Baseline | 10 (10, 10) | 10 (10, 10) | P=0.713 |
| 24-h | 10 (10, 10) | 10 (10, 10) |  |
| QoR2: Been able to enjoy food | | | |
| Baseline | 10 (10, 10) | 10 (10, 10) | P=0.114 |
| 24-h | 9 (5, 10)* | 10 (9, 10)* |  |
| QoR3: Feeling rested | | | |
| Baseline | 10 (8, 10) | 9.5 (7, 10) | P=0.078 |
| 24-h | 7 (5, 9)* | 8.5 (6, 10)* |  |
| QoR4: Have had a good sleep | | | |
| Baseline | 7.3±2.7 | 7.3±2.3 | P=0.066 |
| 24-h | 5 (2, 7) | 7 (4, 10) |  |
| QoR5: Able to look after personal toilet and hygiene unaided | | | |
| Baseline | 10 (8, 10) | 10 (8, 10) | P= 0.309 |
| 24-h | 7.5 (5, 10) | 8 (5, 10) |  |
| QoR6: Able to communicate with family or friends | | | |
| Baseline | 10 (10, 10) | 10 (10, 10) | P=0.214 |
| 24-h | 10 (8, 10) | 10 (10, 10) |  |
| QoR7: Getting support from hospital doctors and nurses | | | |
| Baseline | 10 (10, 10) | 10 (10, 10) | P=0.112 |
| 24-h | 10 (9, 10) | 10 (10, 10) |  |
| QoR8: Able to return to work or usual home activities | | | |
| Baseline | 8 (6, 10) | 10 (8, 10) | P= 0.861 |
| 24-h | 3.5 (2, 7) | 5 (2, 8) |  |
| QoR9: Feeling comfortable and in control | | | |
| Baseline | 8 (7, 10) | 8 (6,10) | P=0.019 |
| 24-h | 3.5 (2, 8)* | 7.5 (4,9)* |  |
| QoR10: Having a feeling of general well-being | | | |
| Baseline | 8 (6,10) | 8.5 (6,10) | P=0.901 |
| 24-h | 5 (4,9) | 7.5 (4,10) |  |
| QoR11: Moderate pain in last 24 hours | | | |
| Baseline | 5 (3,7) | 7 (4,9) | P=0.250 |
| 24-h | 2 (4,6)* | 5 (3,8)* |  |
| QoR12: Severe pain in last 24 hours | | | |
| Baseline | 10 (9,10) | 10 (9,10) | P=0.022 |
| 24-h | 8 (5,10)* | 10 (8,10)* |  |
| QoR13: Nausea or vomiting in last 24 hours | | | |
| Baseline | 10 (10, 10) | 10 (10, 10) | P= 0.563 |
| 24-h | 9 (8,10) | 10 (7,10) |  |
| QoR14: Feeling worried or anxious in last 24 hours | | | |
| Baseline | 5 (3, 10) | 8 (4, 10) | P=0.185 |
| 24-h | 7.5 (3, 10) | 6 (3, 10) |  |
| QoR15: Feeling sad or depressed in last 24 hours | | | |
| Baseline | 8 (5, 10) | 10 (6, 10) | P=0.808 |
| 24-h | 7.5 (5, 10) | 10 (7, 10) |  |

Values are presented as median (1Q, 3Q)

QoR= quality of recovery

*means a difference between the erector spinae plane block and superior trunk block with a *P*< 0.05.
